# Percutaneous Left Atrial Appendage Occlusion—Current Evidence and Future Directions

**DOI:** 10.3390/jcm12237292

**Published:** 2023-11-24

**Authors:** Johannes Rotta detto Loria, Steffen Desch, Janine Pöss, Katharina Kirsch, Holger Thiele, Marcus Sandri

**Affiliations:** Department of Cardiology, Heart Center Leipzig at University of Leipzig, 04289 Leipzig, Germany; johannes.rottadettoloria@medizin.uni-leipzig.de (J.R.d.L.); holger.thiele@medizin.uni-leipzig.de (H.T.)

**Keywords:** left atrial appendage occlusion, atrial fibrillation, anticoagulation, ischemic stroke, percutaneous closure

## Abstract

Over the past two decades, percutaneous left atrial appendage occlusion (LAAO) has proven to be a viable alternative to oral anticoagulation (OAC) for stroke prevention in patients with atrial fibrillation (AF), in particular in those patients who are at increased risk for stroke and bleeding complications. This systematic review provides a comprehensive evaluation of anatomical features, patient selection, procedural planning and execution, complications, medical treatment following the procedure, and contemporary outcome data.

## 1. Introduction

Atrial fibrillation (AF) is the most common sustained cardiac arrhythmia worldwide, with a projected increase in prevalence among the elderly population within the next decades [1]. Accounting for approximately 20–30% of ischemic strokes, AF contributes significantly to the morbidity and mortality of these patients and hence to the economic health care burden [2]. Depending on the risk of ischemic stroke, most frequently estimated by the CHA_2_DS_2_-VASc score, long-term oral anticoagulation (OAC) is the standard of care for stroke prevention [2,3].

In patients with AF, the source of cardioembolic stroke in >90% of the cases is thrombi in the left atrial appendage (LAA) as detected by transesophageal echocardiography (TEE) or autopsy studies [4,5]. Blood stagnation, the absence of atrial contractility in patients with AF, and the partly multi-lobed LAA morphologies with highly trabeculated endocardium are major factors contributing to thrombus formation [6,7].

There are several factors limiting the use of OAC. These are, on the one hand, patient-dependent, including bleeding complications, renal dysfunction, cognitive impairment, and fall risk; on the other hand, they are caused by the practitioner by abstaining or discontinuing OAC because of unfavorable patient characteristics. Even though there is a clear recommendation for the use of OAC in the majority of patients with AF, registry data show that less than 50% of patients with known AF are on adequate OAC [8]. Moreover, adherence to OAC is low, and discontinuation is common. Data from patients on direct oral anticoagulants (DOACs) showed a 1-year adherence of <80% [9,10].

These factors have prompted the development of a nonpharmacologic alternative for stroke prevention. Over the past 20 years, the field of percutaneous LAAO has emerged as a safe and effective therapy to reduce stroke risk.

Because of safety concerns regarding the LAAO procedure, a comprehensive clinical interrogation spanning 2 decades, and two randomized controlled trials (RCTs) were mandated before Food and Drug Administration (FDA) approval was ultimately granted for the WATCHMAN™ device (Boston Scientific, Boston, MA, USA) in 2015. Both the PROTECT-AF (NCT00129545) and the PREVAIL trial (NCT01182441) compared the WATCHMAN™ 2.5 device to warfarin [11,12]. The PRAGUE-17 trial (NCT02426944) compared LAAO with the AMPLATZER™ Amulet™ occluder (Abbott, IL, USA) and the WATCHMAN™ device to DOAC [13]. All trials demonstrated non-inferiority in the reduction of stroke and systemic embolism in LAAO compared to OAC. Notably, all patients had to be eligible for OAC. A meta-analysis of those three RCTs with a combined total of 1516 patients comparing LAAO to OAC showed favorable outcomes for LAAO concerning hemorrhagic stroke, all-cause mortality, cardiovascular mortality, and non-procedure-related major bleeding [14].

Besides data from the RCTs, “real-world” experience was collected in large registries mandated by the FDA as part of the approval process. The Continued Access to PROTECT AF (CAP) and Continued Access to PREVAIL (CAP2) registries evaluated the long-term safety and efficacy of the WATCHMAN™ device in a total of 1144 patients [15]. The EWOLUTION registry included 1025 patients from European centers treated with the WATCHMAN™ device [16]. The National Cardiovascular Data Registry (NCDR)-LAAO, which includes 38,158 patients, is the largest registry to date [17]. In summary, data from the pivotal RCTs and the prospective registries demonstrated increasing procedural success over the years, defined as delivery and release of the device into the LAA, starting at 90.9% in PROTECT-AF and reaching 98.3% in the NCDR-LAAO registry. Conversely, serious procedure-/device-related events decreased significantly, with 8.7% reported in PROTECT-AF dropping to 2.16% documented in the contemporary NCDR-LAAO registry. Notwithstanding the growing body of evidence on LAAO, important questions especially concerning adequate patient selection and optimal antithrombotic regimen following LAAO remain unanswered. Ongoing trials are discussed in the section “Future Directions”.

With the rising prevalence of AF and the growing need for stroke prevention, economic evaluations become increasingly important in view of globally inflating health care costs. When comparing LAAO as a one-time procedure to the lifelong administration of OAC, several studies have estimated cost-effectiveness in favor of LAAO [18,19,20]. However, a systematic review including 12 studies that aimed to assess the cost-effectiveness of LAAO found shortcomings in the methodological quality of these studies, raising concerns about the robustness of the results [21]. The forthcoming expiry of DOAC drug patents will have—at least in patients eligible for OAC—an impact on health economic arguments.

Devices for LAAO

**Figure 1:**
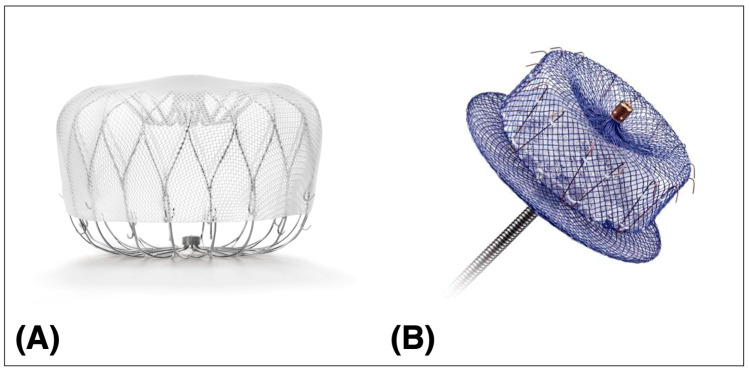
Percutaneous LAAO devices. (**A**) WATCHMAN™ FLX, (**B**) AMPLATZER™ Amulet™. LAAO left atrial appendage occlusion.

Currently, two commercially available devices have received approval by the FDA for LAAO in selected patients (Figure 1). Other devices are available for investigational use in the US only, whereas in Europe many have a CE mark and can be used clinically. In general, the catheter-based devices for LAAO are categorized in a plug type like the WATCHMAN™, WaveCrest^®^ (Biosense Webster, Irvine, CA, USA), and CLAAS^®^ (Conformal Medical, Nashua, NH, USA) devices. The pacifier type includes the AMPLATZER™ Amulet™, the Ultraseal (Cardia, Eagan, MN, USA), and the LAmbre™ devices (Lifetech Scientific, Shenzhen, China). The two most commonly deployed devices are presented here in detail.

The plug-based WATCHMAN™ FLX device consists of a self-expanding nitinol frame with 10 fixation anchors covered by a polyethylene terephthalate cap. It is a technical evolution of the initial WATCHMAN™ 2.5 device. The device is available in five sizes and must be selected based on periprocedural TEE measurements of the LAA ostium and device landing zone to achieve approximately 10–30% compression. It can be recaptured and repositioned if needed.

The AMPLATZER™ Amulet™ LAA occluder is a self-expanding disc- and lobe-based device consisting of a flexible braided nitinol mesh construction. It is a second-generation redesign of the AMPLATZER™ Cardiac Plug. The distal lobe with stabilizing wires is placed approximately 10 mm distal to the LAA orifice in the so-called “landing zone”. The proximal disc then covers the orifice completely. The device is available in eight different sizes. Recapture and repositioning are also possible with this system.

## 2. Patient Selection

### 2.1. Patients Who Should Be Considered

With the current data available, professional societies from the United States and Europe have both stated a class IIb level of evidence B recommendation for LAAO in patients with AF and contraindications for long-term OAC. Criteria for patients suitable for LAAO are summarized in Table 1 [2,3].

Patients evaluated for LAAO should meet the definition criteria of AF in accordance with the current American and European guidelines.

Candidates should be at increased risk of stroke. The inclusion criteria regarding the predicted stroke risk varied across the RCTs: in the PROTECT-AF trial, a CHADS_2_-Score ≥ 1; in PREVAIL and Amulet IDE, a CHADS_2_-Score ≥ 2 was mandated. Ultimately, the PINNACLE FLX trial adopted the contemporary risk stratification with a CHA_2_DS_2_-VASc score of ≥2 for men or ≥3 for women. The latter definition is currently considered as increased stroke risk.

Furthermore, patients should have clinical risk factors for bleeding with OAC (e.g., HAS-BLED score ≥ 3) or other contraindications to OAC. Although there is currently limited data on OAC-ineligible patients, the practice of performing LAAO in patients who are unsuitable for long-term OAC is widely adopted.

Common with other cardiac interventions, life expectancy should exceed a minimum of 1 year, and there should be a benefit in quality of life. A retrospective analysis of a multicentric LAAO registry identified elderly age, lower body mass index (BMI), diabetes, heart failure, and lower glomerular filtration rate as features associated with early death (within 1 year of the procedure). The presence of ≥4 features increased the estimated early death rate to nearly 50% [23]. This finding should—despite the limitations inherent to a retrospective analysis—stimulate a shared operator–patient discussion to elucidate the benefit from the procedure in this frail high-risk cohort.

### 2.2. Patients Who Should Not Be Considered

In general, patients who underwent prior surgical LAA exclusion or those with pre-existing LAA thrombus are not suitable for LAAO. The latter have been excluded from the major RCTs. In the NCDR registry, LAA or LA thrombus was detected on the day of the procedure in 2.25% of patients in the overall cohort, 0.75% of those with successful implantation, 2.25% of those with aborted procedures, and 48.8% of those with procedures canceled before vascular access [17]. A systematic review of all published cases of LAAO in the presence of LAA thrombus demonstrated feasibility of LAAO procedure with some modifications to the standard (using a cerebral protection device and minimizing guidewire and catheter manipulation of the LAA) and if the thrombus was located distally in the LAA body [24]. Direct LAAO versus deferred LAAO after intensification of antithrombotic therapy was compared in a retrospective observational registry including 126 patients with LAA thrombus on preprocedural imaging. Intensification of antithrombotic therapy led to initial thrombus resolution in 60% of the cases but was associated with a relatively high bleeding rate. Direct LAAO, on the contrary, was feasible and safe [25]. However, expert consensus advocates against performing LAAO in patients with known LAA thrombus [26].

Patients with LAA anatomy prohibitive of safe occluder device placement should be excluded. Moreover, LAAO is contraindicated in patients with AF and concomitant relevant mitral stenosis and others for whom long-term OAC is required (e.g., mechanical prosthetic heart valves). Combined procedures such as pulmonary vein isolation (PVI) or other structural heart interventions like transcatheter aortic valve replacement (TAVR) are increasingly performed simultaneously with LAAO, and RCTs for these constellations are underway, e.g., OPTION (NCT03795298; OAC vs. WATCHMAN™ FLX following PVI), WATCH-TAVR (NCT03173534; TAVR with OAC vs. TAVR with WATCHMAN™ LAAO in patients with AF), and WATCH-TMVR (NCT04494347; transcatheter mitral valve repair with WATCHMAN™ LAAO in patients with AF). However, with results pending from these RCTs, combined procedures with LAAO are not routinely recommended.

## 3. Imaging

### 3.1. Anatomy and Function of the LAA

The LAA is a thin-walled remnant of the embryological left atrium. It communicates through an oval-shaped ostium with the left atrium and is laterally confined by the left lateral ridge, an infolding of the atrial wall, and the left upper pulmonary vein [27]. The medial border of the ostium is delimitated by the left circumflex artery. Both structures are important landmarks for procedural planning and device sizing.

The anatomic structure of the LAA is highly variable and has been divided into four morphologies: (1) “windsock”, (2) “chicken wing”, (3) “cactus”, and (4) “cauliflower” [28]. It has been shown that the different LAA types vary in the prevalence rate of cerebrovascular events with higher stroke risk in multi-lobed morphologies like the “cactus” and “cauliflower” types [29,30]. Moreover, in complex morphologies, adequate closure can be challenging, and thorough preprocedural planning is required.

The LAA serves mechanical and neurohormonal functions. The myocardium of the LAA produces atrial and brain natriuretic peptides (ANPs and BNPs). Natriuretic peptides regulate the cardiovascular system exerting natriuretic, diuretic, and vasodilatory effects. Atrial and ventricular remodeling is characterized by elevated levels of the natriuretic peptides. The secretion of proinflammatory and prothrombotic proteins from the LAA endocardium may promote, along with structural remodeling, thrombus formation [31]. The impact of LAAO on the neurohormonal functions and physiological regulation is currently under investigation [32,33,34].

### 3.2. Baseline Imaging

Preprocedural imaging ought to address (1) exclusion of pre-existing LAA thrombus, (2) assessment of LAA morphology including orifice dimensions and depth, and (3) adequate selection and sizing of a suitable closure device.

Two-dimensional (2D) TEE is the most employed pre-imaging modality. The TEE probe should be placed at the mid-esophagus with LAA visualization and image acquisition at 0°, 45°, 90°, and 135°. When in doubt, application of ultrasonic enhancement agents may be used for reliable exclusion of LAA thrombus.

Measurements of the LAA “landing zone” and depth dimensions are obtained ideally during the maximal filling of the LAA (at left ventricular systole). Three-dimensional TEE should be performed to visualize the shape of the orifice. Different sizing algorithms exist for the two commercially available closure devices. The manufacturer’s general information should be consulted for detailed description of orifice and depth measurements.

Invasiveness, the need for fasting, and sedation are major disadvantages of LAAO. Relative and absolute contraindications to TEE need to be evaluated individually.

Dedicated cardiac computed tomography (CT) can also be used as a non-invasive preprocedural imaging tool which persuades with high spatial resolution, access route planning, and ruling out LAA thrombus when imaging protocols are adapted. Multiplanar reconstructions and 3D capabilities allow detailed characterization of the LAA morphology and the surrounding structures for accurate device selection/sizing. LAA dimensions should be assessed preferably at late atrial diastole. Possible advantages concerning improved device selection accuracy and procedural efficiency compared to 2D TEE have been reported. Impaired renal function or contrast allergy limits the use of this imaging modality. Detailed information on CT acquisition techniques for LAAO can be found in a recently published expert recommendations document [35].

### 3.3. Periprocedural Imaging

Key elements of procedural imaging are (1) ruling out LAA thrombus, (2) guiding the transseptal puncture (TSP), (3) visualization of device implantation and release, and (4) recognition of intraprocedural complications.

The combination of TEE and fluoroscopy is the standard approach for intraprocedural imaging, as most operators are well experienced with these methods. Guidance for TSP and sheath positioning is best achieved in a bicaval view. Standardized TEE views at 0°, 45°, 90°, and 135° and a right anterior oblique caudal angiographic projection are typically utilized for device release. Specific imaging criteria for successful LAA closure device implantation and release have been proposed by the device manufacturers (Table 2).

In recent years, intracardiac echocardiography (ICE) has gained an emerging role as an alternative for procedural guidance. The most common modality in use is the phased-array ICE. It consists of a transducer mounted on the distal tip of a steerable catheter. The catheter is advanced, as usual, through femoral venous access. Standard echocardiographic views are then obtained from the right atrium and ventricle. However, LAA visualization can be impaired from these positions, requiring the passage of the ICE probe through the interatrial septum into the LA. Advantages of this method are a reduction in fluoroscopy time, risk of esophageal complications, and the lack of need for a second operator. The acclaimed advantage of performing ICE only under conscious sedation may be depreciated, since procedural TEE is increasingly performed, especially in European centers, without general anesthesia. The costs of the ICE probe, lesser validated device release criteria, and increased procedural complexity when placing the ICE probe in the LA are considered disadvantageous for this imaging modality. An in-depth description of the use of ICE in LAAO has been published elsewhere [36]. In general, the use of fluoroscopy alone without TEE or ICE guidance is not recommended.

### 3.4. Postprocedural Imaging and Device Surveillance

An overview of postprocedural imaging is outlined in Table 3. Pericardial effusion should be ruled out immediately after LAAO in the catheterization laboratory. The pre-discharge 2D TTE should assess device position and rule out pericardial effusion again. According to expert recommendations, a follow-up visit at 45 to 90 days after LAAO for assessment of postprocedural complications, e.g., peri-device leak and device-related thrombus, should be scheduled [26]. Both TEE and CT are appropriate imaging modalities in that matter. In case of specific findings or adverse clinical events, the additional follow-up timing is to be determined individually.

## 4. Procedural Aspects

Rather than giving a complete description of the LAAO procedure, only general aspects are discussed in this section, as catheterization laboratories may differ in their routines. Most of the recommendations are based on the SCAI/HRS Expert Consensus Statement on Transcatheter Left Atrial Appendage Closure [26]:

- The widely implemented TEE guidance for LAAO is the main reason for the utilization of general anesthesia in the US. As a feasible and safe alternative, many European centers routinely perform LAAO with a moderate conscious sedation protocol resulting in a reduction in procedural time, hospital stay, and costs [37,38].
- Vascular access is preferably achieved by ultrasound-guided puncture of the right femoral vein.
- Therapeutic anticoagulation is attained by administration of unfractionated heparin. Activated clotting time (ACT) should range between 250 and 300 s. Periodical ACT monitoring is obligatory. After completion of the intervention, administration of protamine may be considered if hemostasis is not achieved by other means (e.g., manual compression, suture, etc.).
- Coaxial orientation of the access sheath with the LAA is the purpose of a correct TSP. Typically, an inferior and posterior septal position on the interatrial septum is chosen. Specific anatomy may require a different TSP site; preprocedural imaging can help determine the location of the TSP. Passage through a patent foramen ovale should be avoided as it may not result in coaxiality of the device with the LAA. TEE guidance for TSP and sheath positioning is best achieved in a bicaval view.
- LAA dimensions vary depending on the hydration status. For correct device sizing, LAA measurements should be performed when mean LA pressure is ≥12 mmHg.
- Entry into the LAA should be as atraumatic as possible to reduce the risk of perforation/pericardial effusion. The use of a pigtail catheter is advocated.
- LAAO device deployment is performed according to the manufacturer’s instruction. Established release criteria for the two available devices should be considered (Table 2).
- Assessment of pericardial effusion should be performed at the beginning and end of the LAAO procedure.
- Given the risk of periprocedural stroke, a neurological assessment should be performed after anesthesia/sedation.
- Administration of appropriate endocarditis prophylaxis for 6 months after device implantation.

## 5. Intraprocedural Complications

The device-related complication rate was relatively high in the first published experience (8.7% in PROTECT-AF) but had already dropped significantly in the ensuing RCT investigating the WATCHMAN™ device (4.4% in PREVAIL) [11,12]. In a head-to-head comparison of the AMPLATZER™ Amulet™ and the outdated WATCHMAN™ 2.5 device (Amulet IDE trial), patients treated with the Amulet™ device had significantly more procedure-related complications (4.5 vs. 2.5%) [34]. With increasing operator experience, technical evolution of the closure devices, and procedural improvements, the complication rate has been further mitigated. Real-world data illustrated by the NCRD-LAAO registry with over 38,000 patients show that the incidence of major complications is roughly 2% [17]. Notwithstanding a low complication rate, the following complications should receive special attention.

### 5.1. Pericardial Effusion

Pericardial effusion accounted for the majority of the adverse events reported in the RCTs [11,12,39]. Initially ranging around 5%, the incidence of pericardial effusion has dropped to below 1.5% because of device modifications and procedural improvements. The etiology of pericardial effusion is in most cases trauma to the thin-walled LA, LAA, or pulmonary veins. Perforation may occur during TSP, advancement of sheaths, and guidewires or in the process of delivering/repositioning the occlusion device. Pericardial effusion may result in tamponade, marking a life-threatening complication. Late pericardial effusion is an infrequent phenomenon.

### 5.2. Management of Pericardial Effusion/Tamponade

Continued monitoring of vital signs and intraprocedural imaging of the pericardial space are paramount to early diagnosis of pericardial effusion. In the NCDR-LAAO registry, 528 of 38,158 patients (1.39%) suffered from pericardial effusion requiring further treatment. Of these 528 patients, 437 (83%) underwent pericardiocentesis, and 91 patients (17%) needed cardiac surgery [17]. Acute management of pericardial effusion includes interventional pericardiocentesis with insertion of a pigtail catheter, drainage, and autotransfusion in cases of active pericardial bleeding. In refractory situations, e.g., overt perforation, urgent surgery may be discussed.

Immediate availability of pericardiocentesis kits and surgical teams are critical institutional requirements to enhance periprocedural safety.

### 5.3. LAAO Device Embolization

Device embolization is a rare complication. Data from the RCTs outline a low very low incidence: 0% for the second-generation WATCHMAN™ FLX (PINNACLE trial) and 0.7% for the AMPLATZER™ Amulet™ (Amulet IDE trial) [39,40]. Real-world experience from the NCDR-LAAO registry shows an incidence of 0.07% [17]. Approximately two thirds of embolizations occur during implantation and the other third later in time—often detected as incidental finding. The embolized device may be located in the LA, the left ventricle, or in the aorta. Device under- or oversizing as well as too proximal implantation may contribute to device migration.

### 5.4. Management of Device Embolization

Depending on the location of the embolized device, different strategies for device retrieval may be implemented. Device entanglement with the mitral valve apparatus or lodging in the left ventricle are destined to be surgically removed. A percutaneous approach by snaring the device may be attempted, if the device is still in the LA or has migrated to the aorta.

### 5.5. Periprocedural Strokes

Periprocedural stroke rates have decreased significantly with operator experience and procedural refinements. The NCDR-LAAO registry reports an incidence of ischemic and hemorrhagic stroke of 0.12% and 0.01%, respectively [17]. Sources of periprocedural stroke may be pre-existing thrombus, air embolization, or thrombus formation on the interventional equipment.

### 5.6. Management of Periprocedural Stroke

The various causes for stroke can be constrained by the following measures: thorough pre-imaging of the LAA for thrombus exclusion; meticulous preparation of the device with rigorous purging, flushing, and deairing of the equipment; maintaining an adequate procedural anticoagulation (ACT > 250 s with frequent controls); continued TEE observance of possible thrombus formation during the procedure. Patients in whom clinically overt stroke has occurred should undergo expeditious neurological assessment and treatment according to the local standard.

## 6. Postprocedural Complications

### 6.1. Device-Related Thrombosis

**Figure 2:**
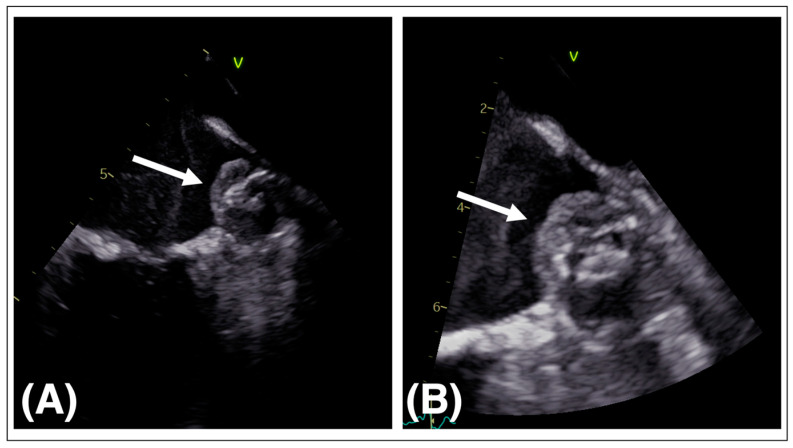
Device related thrombosis. (**A**,**B**) 2D TEE of the LA after LAAO and visualization of device related thrombosis on the atrial surface of the WATCHMAN™ device (white arrows). TEE transesophageal echocardiography, LA left atrium, LAAO left atrial appendage occlusion.

Device-related thrombosis (DRT) relates to thrombus formation on the atrial surface of the LAAO device after implantation (Figure 2). Data from the RCTs and registries suggest an association of DRT and increased risk of stroke. This is problematic for two reasons: LAAO as an intended prophylactic treatment for stroke reduction becoming itself a hazard for thromboembolic events and secondly OAC as a recommended treatment for thrombus resolution in a cohort in which OAC was sought to be avoided.

The reported incidence of DRT ranges between 2 and 5% with most cases detected within 180 days after device deployment [11,12,17]. DRT is usually detected by TEE in multiple views or by CT showing hypoattenuated thickening (HAT). 

A study including 1739 patients with data from the pivotal RCTs PROTECT-AF and PREVAIL, as well as from the successional registries CAP and CAP2, demonstrated that the presence of DRT was significantly associated with higher rates of ischemic stroke/systemic embolism (adjusted hazard ratio, 3.9; 95% CI, 2.3–6.8; *p* < 0.001). Rates of cardiovascular or all-cause mortality did not differ significantly for patients with and without DRT [41]. The Amulet observational study enrolled 1088 patients, of which 18 had DRT. Patients with DRT had significantly higher stroke rates than patients without DRT within the 1-year follow-up (18.3% vs. 2.6%, *p* < 0.01). Also, in this study, rates of all-cause mortality for patients with and without DRT were not different (14.4% vs. 8.3%, *p* = 0.64) [42]. A meta-analysis comparing 7689 patients with and without DRT substantiated the increased risk of ischemic stroke in patients with DRT (13.2% vs. 3.8%, *p* < 0.001) [43]. The multinational EUROC-DRT registry including 156 patients with DRT revealed a high risk of mortality (20.0%) and ischemic stroke (13.8%) in those patients within the 2-year follow-up [44]. Several studies showed that the various antithrombotic regimens after LAAO did not have an impact on the occurrence of DRT [41,43,45,46]. 

Several risk factors for the development of DRT have been suggested, including large LAA dimensions, permanent AF, LAAO device implantation well beneath the left lateral ridge hence creating a “neo-appendage”, and pre-existing LAA thrombus and coagulation disorders [43,45,47,48]. A higher frequency of DRT has been reported in patients with peri-device leaks [46]. Whether one of the two LAAO devices is more prone to DRT remains unclear. In the Amulet IDE trial, the Amulet™ device had a numerical advantage compared to the WATCHMAN™ 2.5 device (3.3% vs. 4.5%) [39]. Design changes in the next-generation WATCHMAN™ FLX device were intended to reduce thrombogenicity. The DRT rate for this device was 1.7% after 1 year as reported in the PINNACLE FLX trial [40]. DRT should be treated with OAC. Treatment strategies include administration of vitamin K antagonists (VKA) or DOAC for 8–12 weeks (target international normalized ratio of 2–3) in patients not already on OAC therapy. Intensification of antithrombotic therapy (international normalized ratio 2.5–3.5) may be considered in patients already on VKA [49]. Data from the EUROC-DRT registry report significantly higher incidence of stroke and mortality after LAAO in patients with residual DRT compared to patients with DRT resolution after 1 year (stroke: 17.6% vs. 6.5%, *p* = 0.09; mortality: 15.0% vs. 1.4%, *p* = 0.01) [44]. Repeat imaging for assessment of thrombus resolution and decision on eventual cessation of OAC are recommended at a 45- to 90-day interval. In summary, DRT after LAAO is an uncommon finding but is associated with a manifold increase in ischemic stroke events. Whether DRT is directly responsible for adverse events or other comorbidities, e.g., hypercoagulable disease, remains unclear.

### 6.2. Peri-Device Leaks

**Figure 3:**
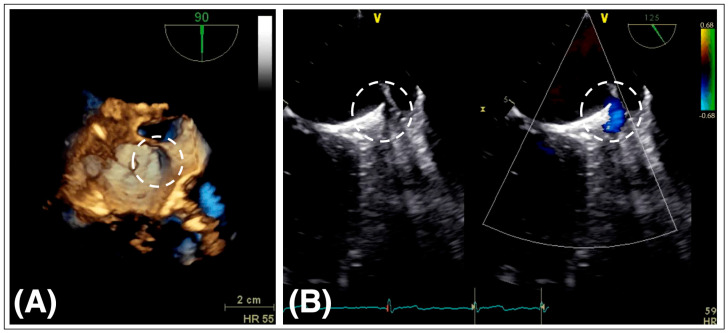
Peri-device leaks. (**A**) 3D TEE of the left atrium after LAAO with AMPLATZER™ Cardiac Plug and residual PDL (dashed circle), (**B**) left: 2D TEE after LAAO with incomplete sealing and resulting PDL (dashed circle), right: residual blood flow around the LAAO device edge into the left atrium as seen by color Doppler (dashed circle), TEE transesophageal echocardiography, LAAO left atrial appendage occlusion, PDL peri-device leak.

Peri-device leaks (PDLs) are defined as residual blood flow around the LAAO device edging into the LAA body (Figure 3). Two mechanisms may contribute to the formation of PDL. The LAA orifice is predominantly elliptic, while the LAAO devices are round [50]. Non-coaxially deployed LAAO devices with resulting malalignment may additionally contribute to this geometrical mismatch [51].

Registry data indicate that device oversizing may lower the odds of residual leaks without an increase in the rate of pericardial effusion [52].

PDLs can be detected either by TEE with color Doppler imaging or contrast-enhanced CT. Depending on the device and the imaging modality used for detection, PDLs are a common finding after LAAO. The reported incidence ranges between 11% and 57% [11,39]. Depending on the residual jet size, PDLs have been graded in none, minimal (jet diameter < 1 mm), mild (1–3 mm), moderate (3–5 mm), and severe (5 mm). The incidence of severe leaks in this study was 0.6% [49]. Given the arbitrary nature of this definition, grading of leak size across the different studies is heterogenous. In a substudy of the Amulet IDE trial, the Amulet™ occluder had significantly fewer severe (>5 mm) PDLs than the WATCHMAN™ device at 45 days (1.1 vs. 3.2%, *p* < 0.001) and 12 months (0.1 vs. 1.1%, *p* < 0.001) [39].

In view of conflicting data, the clinical impact of PDLs remains unclear. In general, the rate of thromboembolic events after LAAO is low; thus, assessing an independent effect of PDLs on adverse events would require a very large sample size. A secondary analysis of the PROTECT-AF trial found no significant difference in adverse event rates irrespective of the leak size (<1, 1–3, and >3 mm) [53]. A multicenter, retrospective study including 108 patients with PDLs found that the incidence of transient ischemic attack or stroke in patients with PDLs was significantly higher compared with patients without PDLs (8.3% vs. 2.7%, *p* = 0.005), irrespective of PDL size [54]. In contrast, the NCDR-LAAO registry demonstrated an association with increased risk of transient ischemic attack, stroke, and systemic embolization (adjusted HR: 1.152; 95% CI: 1.025–1.294) and major bleeding (adjusted HR: 1.110; 95% CI: 1.029–1.199) at 1 year with small PDLs < 5 mm. Large leaks (>5 mm) compared to small or no leaks were not associated with a higher rate of adverse events. However, large leaks were infrequent (0.7%), and the study may have been underpowered to assess an association between large leaks and adverse events [55]. In a subgroup analysis of the Amulet IDE trial, the presence of PDLs > 3 mm was associated with a higher risk of the composite endpoint of ischemic stroke, systemic embolization, or cardiovascular death (8.1% vs. 4.7%; adjusted HR: 1.66; 95% CI: 1.02–2.69; *p* = 0.04) [56]. There is, however, a broad consensus about the failure of LAAO when PDLs > 5 mm persist. In this case, the continuation of OAC is customary. The feasibility of other interventional approaches (coils or plug devices) to seal the PDL have been demonstrated on an anecdotal level [57,58].

## 7. Antithrombotic Regimen after LAAO

The initial specific postprocedural treatment protocols were derived from the pivotal RCTs recommending a dual therapy with warfarin and aspirin for 45 days after LAAO, followed by dual antiplatelet therapy (DAPT) with aspirin and clopidogrel for 6 months, and finally aspirin alone if no DRT or PDL was present. Since then, there has been great variability regarding antithrombotic medication and duration after LAAO.

In adherence to current guideline recommendations for the medical prevention of thromboembolic events in AF, in which DOACs are recommended in preference to VKA, changes to the discharge antithrombotic strategy have been made accordingly. In the four pivotal RCTs of DOACs in comparison to VKA in patients with non-valvular AF, the risk of intracerebral hemorrhage was significantly lower with all DOACs. Risk of major gastrointestinal bleeding was higher when treated with Dabigatran 150 mg and Rivaroxaban and Edoxaban 60 mg. Risk of major bleeding was reduced with most DOACs except treatment with Dabigatran 150 mg and Rivaroxaban [59,60,61,62]. A network meta-analysis comprising 23 randomized trials involving a total of 94656 patients showed that Dabigatran 150 mg and Rivaroxaban had a similar risk of major bleeding but were associated with a higher risk of gastrointestinal bleeding compared to VKA. Apixaban 5 mg bidaily was rated best for most outcomes [63]. The favorable safety–effectiveness profile of Apixaban in patients with AF for stroke prevention was confirmed in several real-world studies and meta-analyses [64,65,66,67].

In contemporary US practice, roughly 21% of patients after LAAO are discharged with DOAC and aspirin. Adverse event rates were comparable to warfarin and aspirin. The sole administration of warfarin or DOAC without concomitant aspirin was associated with lower risk of adverse events. This was mainly explained by fewer bleeding complications [68].

Avoiding the continuation of OAC following LAAO in patients who are poor candidates for OAC in the first place was another consideration for protocol deviations. Outside the US, it is common practice to discharge patients after LAAO on DAPT [16]. Evidence from the Amulet IDE trial, from prospective registries, and a large meta-analysis showed no significant difference in DAPT versus OAC after LAAO with respect to rates of adverse events and postprocedural complications [16,39,69]. Yet, a randomized head-to-head comparison of OAC and DAPT is still warranted. Data on early single antiplatelet therapy after LAAO are limited [70,71,72]. Data for patients with extremely high risk for bleeding are even more scarce. Several registries included patients discharged without any antithrombotic therapy. The prospective Amulet observational study including 1088 patients treated with the AMPLATZER™ Amulet™ device reported an increasing proportion of patients without antithrombotic medication from 2.0% at discharge rising to 21.5% after 2 years [73]. After 1 year, there were neither cases of ischemic stroke or systemic embolism nor DRT. Major bleeding events were reported in 13% of these patients [74]. In the EWOLUTION registry, 1005 patients received the WATCHMAN™ device and were followed for 2 years. Three to six months after LAAO, a total of 126 patients were switched to no antithrombotic therapy. Rates of adverse events in this cohort were low: three strokes, two bleeding events, and one DRT [75]. A large multinational registry including 1082 patients treated both with the AMPLATZER™ and WATCHMAN™ devices reports approximately 15% of these patients were not treated with any antithrombotic agent within 6 months after LAAO. Early discontinuation did not lead to an increased risk of thromboembolic events but to a reduction in bleeding events after a median follow-up of 2 years. One case of DRT was observed [76]. Safety of early discontinuation (at 6 months) of aspirin is being currently investigated in the randomized ASPIRIN-LAAO trial. In summary, data from the observational registries suggest long-term efficacy for LAAO in patients with extremely high risk for bleeding discharged without any antithrombotic medication.

Up to now, the body of evidence is still inadequate to propose an optimal discharge antithrombotic medication. Hence, a patient-tailored regimen depending on the individual bleeding/thrombotic risk, implanted LAAO device, and possible occurrence of postprocedural complications should be determined until results from the ongoing RCTs on this matter are available.

## 8. Further Directions

In the past 20 years, LAAO has proven to be a safe and effective treatment option for patients with AF at increased risk of stroke and contraindications to long-term OAC. With the increasing experience with LAAO and growing implementation into current practice, questions have been raised about alternative patient populations, procedural improvements, and postprocedural antithrombotic therapy. Ongoing trials aim at broadening the clinical indications, ameliorating procedural safety further and eventually strengthening/upgrading the current guideline recommendations.

### 8.1. Patient Selection

The current societal guidelines recommend LAAO for patients with AF who cannot tolerate long-term OAC. This is a peculiar situation, since OAC-ineligible patients were excluded from the RCTs. LAAO has shown favorable clinical outcomes in OAC-ineligible patients in observational studies, yet these results need to be confirmed in RCTs. This evidence gap is being addressed by the COMPARE-LAAO (NCT046676880) and ASAP-TOO (NCT02928497) trials recruiting patients with contraindications to OAC and aiming at demonstrating the superiority of LAAO compared to the standard of care.

The only RCT comparing DOAC to LAAO for OAC-eligible patients to date is the PRAGUE-17 trial. It demonstrated non-inferiority of LAAO to DOAC but was relatively small with only 201 patients per group [13]. With the CATALYST trial (AMPLATZER™ Amulet™ vs. DOAC, NCT04226547) and the CHAMPION-AF trial (WATCHMAN™ FLX vs. DOAC, NCT04394546), two large studies with approximately 3000 patients per trial are on the way. The CLOSURE-AF trial (NCT03463317) will enroll approximately 1500 patients and compare LAAO (all CE-mark-approved LAA closure devices) to the best medical care (antithrombotic regime at the discretion of the investigator).

Furthermore, RCTs for patient groups with specific indications are being currently conducted. The effect of LAAO in patients with AF and prior intracerebral hemorrhage is being assessed in the STROKECLOSE (NCT02830152) and CLEARANCE (NCT04298723) trials. Results from the above-mentioned OPTION, WATCH-TAVR, and WATCH-TMVR trials may expand the LAAO indication to patients undergoing combined procedures, e.g., structural heart interventions (TAVR/transcatheter edge-to-edge repair) or PVI. The benefit of LAAO in patients with AF and end-stage chronic kidney disease is being evaluated in the LAA-KIDNEY trial (NCT05204212).

### 8.2. Imaging

Preprocedural planning imaging is under constant development, especially in the field of cardiac CT. Computational modeling, 3D printing, and fusion/overlay imaging may substantially contribute to optimal LAAO device sizing and positioning.

The increased use of ICE, emerging 3D capabilities, and ongoing studies (ICE WATCHMAN trial; NCT04569734, safety and feasibility of LAAO with the WATCHMAN™ device guided by ICE) will further integrate this imaging modality into current practice for LAAO.

### 8.3. LAAO Devices

Currently, the WATCHMAN™ and the AMPLATZER™ Amulet™ devices are the most commonly selected and studied devices for LAAO. It remains to be seen whether the other devices will add to the procedural armory and can successfully compete with the already available systems or have a justification owing to niche advantages.

### 8.4. Antithrombotic Regimen

In current practice, many deviations have been made to the FDA-approved postprocedural treatment protocols. Evidence for alternative antithrombotic discharge medications is derived primarily from real-world registries. The importance of this issue is reflected by the multitude of RCTs conducted to elucidate the safety and efficacy of the different antithrombotic regimens. A selection of trials on postprocedural antithrombotic strategies is presented in Table 4. The results from these studies will certainly impact the recommendations for discharge medication.

## 9. Conclusions

LAAO has emerged over the past 2 decades as a safe and effective procedure with remarkable procedural success rates for patients with AF unsuitable for long-term OAC. This has been evaluated in the “real-world” data from large registries and the follow-up evidence from landmark trials. Continued development of imaging modalities and accumulated operator and institutional experience, as well as technical advancements in the occlusion devices, have contributed to the increasing implementation of this therapy. However, many questions regarding adequate patient selection, optimal antithrombotic regimen after the intervention, and management of postprocedural complications remain unanswered. Numerous ongoing trials addressing these issues will expand our knowledge and possibly broaden the indication for LAAO in patients with AF and increased risk for stroke and bleeding.

## Figures and Tables

**Table 1:** Patient criteria for LAAO.

2020 ESC and 2019 AHA/ACC/HRS guideline recommendations ^1,2^	“LAA occlusion may be considered for stroke prevention in patients with AF and contraindications for long-term anticoagulant treatment” (Class of recommendation IIb, Level of evidence B).
Meet definition of clinical AF ^1^	Minimum duration of an ECG tracing of AF required to establish the diagnosis of clinical AF is at least 30 s or entire 12-lead ECG.
High thromboembolic risk	CHA_2_DS_2_-VASc ≥ 2 for men. CHA_2_DS_2_-VASc ≥ 3 for women.
Contraindication to long-term OAC ^3^	HAS-BLED score ≥ 3. Risk for major bleeding, especially life-threatening or disabling bleeding due to an “untreatable” source of: 1. Intracranial/intraspinal bleeding (e.g., diffuse amyloid angiopathy, untreatable vascular malformation). 2. Severe gastrointestinal (e.g., diffuse angiodysplasia) pulmonary or urogenital source of bleeding that cannot be corrected. Severe side effects under vitamin K antagonists and/or contraindication for DOAC.
Life expectancy > 1 year	Assessing comorbidities prohibitive to LAAO.
Quality of life benefit	Patient–provider shared decision.

^1^ Adapted from Hindricks et al. [2]. ^2^ Adapted from January et al. [3]. ^3^ Adapted from Glikson et al. [22]. LAAO, left atrial appendage occlusion; ESC, European Society of Cardiology; AHA, American Heart Association; ACC, American College of Cardiology; HRS, Heart Rhythm Society; LAA, left atrial appendage; ECG, electrocardiogram; AF, atrial fibrillation; DOAC, direct oral anticoagulant; OAC, oral anticoagulation.

**Table 2:** Specific imaging criteria for device release.

PASS for WATCHMAN™ FLX	CLOSE for AMPLATZER™ Amulet™
**P**osition: device is distal to or at the ostium of the LAA **A**nchor: fixation anchors engaged/device is stable **S**ize compression: device is compressed 8–20% of original size **S**eal: device spans ostium, all lobes of LAA are covered	**C**losure: At least 2/3 of the device lobe should be distal to the left Circumflex artery on echocardiography **L**obe compression: The device Lobe should be slightly compressed and have good apposition to the left atrial appendage wall **O**rientation: The Orientation of the device lobe must be in line with the axis of the intended landing zone in the left atrial appendage **S**eparation: The disc must be Separated from the lobe **E**lliptical: The disc will have a concave (Elliptical) shape

Adapted from the manufacturer’s instructions for use. LAA, left atrium appendage.

**Table 3:** Postprocedural imaging and device surveillance.

	Immediately after LAAO	Prehospital Discharge	45–90 Day Follow Up
Imaging modality	TTE	TTE	TEE/CT
Question	Pericardial effusion	Pericardial effusion Device embolization	DRT PDL

Adapted from Saw et al. [26]. LAAO, left atrial appendage occlusion; TTE, transthoracic echocardiography; TEE, transesophageal echocardiography; CT, computed tomography; DRT, device-related thrombosis; PDL, peri-device leak.

**Table 4:** Antithrombotic regimen after LAAO in ongoing trials.

Trial Acronym	NCT	N	Strategy
ADALA	NCT05632445	160	Apixaban vs. DAPT after LAAO.
ANDES	NCT03568890	350	DAPT vs. DOAC for 8 weeks.
ASPIRIN-LAAO	NCT03821883	1120	Continuation vs. discontinuation of aspirin 6 months after LAAO.
APPROACH	NCT04550637	200	Apixaban for 12 weeks after LAAO.
CLOSURE-AF	NCT03463317	1512	DAPT after LAAO.
CLEARANCE	NCT04298723	530	DAPT for 3 months after LAAO, followed by aspirin for 12 months. Alternatively, 3 months of NOAC followed by aspirin for 12 months.
CATALYST	NCT04226547	2650	DAPT for 3 months after LAAO.
CHAMPION-AF	NCT04394546	3000	DOAC or DAPT for 3 months after LAAO.
STROKECLOSE	NCT02830152	750	Aspirin ± Clopidogrel for 45 d after LAAO.
LAA-KIDNEY	NCT05204212	430	DAPT for 3 months after LAAO.

LAAO, left atrial appendage occlusion; NCT, national clinical trial; DAPT, dual antiplatelet therapy; DOAC, direct oral anticoagulant.

## Abbreviations

ACT	Activated clotting time
AF	Atrial fibrillation
CT	Computed tomography
DAPT	Dual antiplatelet therapy
DOAC	Direct oral anticoagulant
DRT	Device-related thrombosis
ECG	Electrocardiogram
FDA	Food and Drug Administration
ICE	Intracardiac echocardiography
LA	Left atrium
LAA	Left atrial appendage
LAAO	Left atrial appendage occlusion
NCDR	National Cardiovascular Data Registry
OAC	Oral anticoagulation
PDL	Peri-device leak
PVI	Pulmonary vein isolation
RCT	Randomized controlled trial
TAVR	Transcatheter aortic valve replacement
TEE	Transesophageal echocardiography
TTE	Transthoracic echocardiography
TSP	Transseptal puncture
VKA	Vitamin K antagonist
